# Cardiac troponin T (cTnT) assessment using incompletely filled K _2_ EDTA blood tubes is reliable

**DOI:** 10.31744/einstein_journal/2024CE0613

**Published:** 2024-01-29

**Authors:** Giuseppe Lippi, Gian Luca Salvagno

**Affiliations:** 1 Section of Clinical Biochemistry School of Medicine University of Verona Verona Italy Section of Clinical Biochemistry and School of Medicine , University of Verona , Verona , Italy .

Dear Editor,

Cardiac troponin testing, which includes the measurement of cardiac troponin I (cTnI) or T (cTnT), is the mainstay of diagnosing acute coronary syndrome, especially in non-ST-elevation myocardial infarction (NSTEMI), in which elevated cTnI or cTnT is the only objective parameter. ^( 1 )^ Ensuring quality throughout the testing process is essential for maintaining the diagnosis integrity. The receipt of underfilled samples is common in clinical laboratories, ^( 2 )^ and this could impair the results of some immunoassays. ^( 3 )^ Therefore, this study aimed to investigate the potential impact of underfilled K _2_ EDTA blood tubes on cTnT assay results.

We studied 17 subjects (mean age 46±9 years, 65% females); recruited from the personnel of the clinical laboratory of the University Hospital of Verona (Italy). Blood was drawn through phlebotomy using a 10mL syringe (Plastipak Luer-Lok 10mL Syringe, Becton Dickinson, Madrid, Spain) and a 21-gauge disposable needle (KDL, Nanchang, Jiangxi, China). It was immediately dispensed into four 3.0mL primary blood tubes with spray-dried K _2_ EDTA (5.4mg) (Vacutest, Kima, Arzergrande, Padova, Italy; Lot: XZ3062) at different volumes: 0.5mL (17% filling), 1.0mL (33% filling), 2.0mL (67% filling), and 3.0mL (100%; correct filling), as described earlier. ^( 4 )^ The empty tubes were premarked at different fill volumes through direct comparison with tubes filled with blood using a calibrated pipette. The samples were centrifuged, and the level of cTnT in the K _2_ EDTA plasma was measured using a Roche Cobas E 601 Module (Roche Diagnostics, Basel, Switzerland), following the manufacturer’s instructions. A comprehensive description of this method and its performance has been reported. ^( 5 )^ All volunteers provided informed consent for participation in this study, which was performed in accordance with the Declaration of Helsinki and all relevant local legislation. This study was approved by the Ethics Committee of the University Hospital of Verona (970CESC; July 20, 2016).

**Figure 1:**
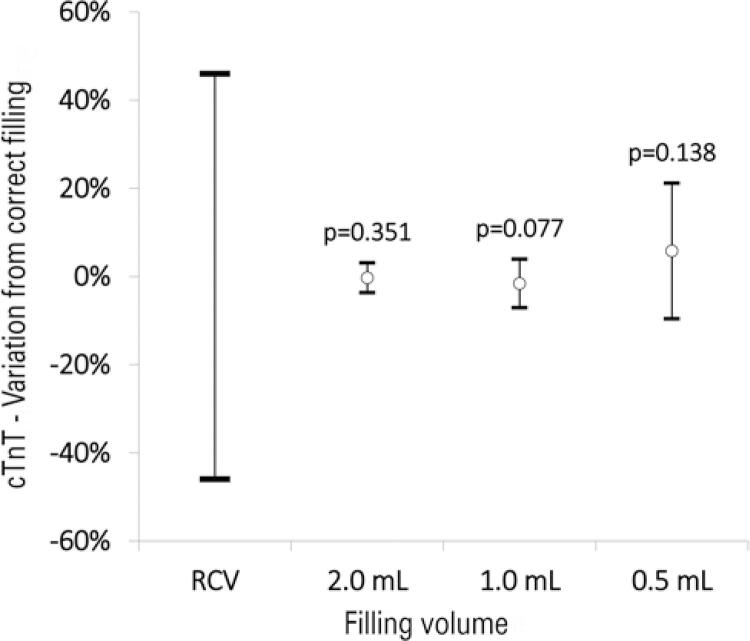
Influence of the filling level of K 2 EDTA blood tubes on cardiac troponin T (cTnT) testing. Differences from the reference (appropriately filled 3.0mL blood tube) are provided above the upper limit of the standard deviation, for comparison with the reference change value. Data are presented as mean±standard deviation

The results are presented in figure 1 . No significant difference in the cTnT levels was observed among the three underfilled samples. Compared with the clinically significant threshold of variation reported as the reference change value, the variation in cTnT values remained well below the acceptable threshold of ±46%. ^( 6 )^

The collection of suboptimal whole blood volumes in K _2_ EDTA blood tubes does not affect the results of the cTnT assays in ostensibly healthy individuals. Therefore, samples underfilled by up to 83% may still be acceptable for cTnT testing using the Roche Cobas E 601 module.
